# Profiling autoantibodies at baseline and during immune checkpoint inhibitor therapy of renal cell carcinoma patients—exploratory results from TITAN-RCC

**DOI:** 10.1016/j.esmoop.2025.105575

**Published:** 2025-08-28

**Authors:** A. Fritzsch, K. Leucht, N. Röber, K. Conrad, R. Rothe, R. Wehner, U. Schumacher, E. Esteban, P. Barthélémy, M. Schmidinger, M. Schmitz, M.-O. Grimm

**Affiliations:** 1 Department of Urology, Jena University Hospital, Friedrich-Schiller University Jena, Jena, Germany; 2 Comprehensive Cancer Center Germany (CCCG), Jena, Germany; 3 Institute of Immunology, Faculty of Medicine Carl Gustav Carus, TUD Dresden University of Technology, Dresden, Germany; 4 Institute for Clinical Chemistry and Laboratory Medicine, University Hospital and Faculty of Medicine, TUD Dresden University of Technology, Dresden, Germany; 5 National Center for Tumor Diseases (NCT), NCT/UCC Dresden, a partnership between DKFZ, Faculty of Medicine and University Hospital Carl Gustav Carus, TUD Dresden University of Technology, and Helmholtz-Zentrum Dresden-Rossendorf (HZDR), Dresden, Germany; 6 German Cancer Consortium (DKTK), Partner Site Dresden, and German Cancer Research Center (DKFZ), Heidelberg, Germany; 7 Center for Clinical Studies, Jena University Hospital, Friedrich-Schiller University Jena, Jena, Germany; 8 Department of Medical Oncology, Central University Hospital Asturias, Oviedo, Spain; 9 Department of Medical Oncology, University Hospital Strasbourg, ICANS, Strasbourg, France; 10 Department of Urology, Medical University of Vienna, Vienna, Austria

**Keywords:** immune-related adverse events, autoantibodies, immunotherapy, immune checkpoint inhibitors, renal cell carcinoma

## Abstract

**Background:**

Immune checkpoint inhibitor (ICI) administration profoundly promotes T-cell-mediated antitumor effects, but also frequently causes the development of immune-related adverse events (irAEs) in cancer patients. Previous studies explored the relevance of autoantibodies as predictive biomarkers for irAE development and treatment response, leading to conflicting results. To gain novel insights, we determined the presence of autoantibodies and their predictive value in ICI-treated renal cell carcinoma (RCC) patients.

**Patients and methods:**

Within the multicenter clinical trial TITAN-RCC (NCT02917772), we prospectively analyzed sera from 170 RCC patients at baseline and various time points during ICI therapy. Immunofluorescence, enzyme-linked immunosorbent assay, and chemoluminescence were applied for the detection of various autoantibody entities. Valuation of titers was defined per individual autoantibody. Statistical analysis was carried out to evaluate correlations between the presence of autoantibodies and irAEs or clinical outcome.

**Results:**

Of 170 ICI-treated RCC patients, 37% had pre-existing autoantibodies. In addition, of 107 autoantibody-negative patients at baseline, 60% showed a new occurrence of autoantibodies during treatment. Patients with pre-existing autoantibodies significantly less often developed irAEs during therapy than autoantibody-negative patients. New occurrence of the majority of autoantibodies during therapy was not significantly linked to irAE incidence. We only found a significant correlation between the presence of anti-thyroid peroxidase/anti-thyroglobulin during maintenance therapy with thyroid dysfunction development. While a significant association between pre-existing autoantibodies and disease control was not observed, we found a significant correlation between the occurrence of novel autoantibodies during therapy and a higher disease control rate for RCC patients. Moreover, patients with treatment-associated irAEs significantly more often achieved disease control.

**Conclusions:**

Our findings revealed that neither the presence of autoantibodies at baseline nor the new occurrence of the majority of investigated autoantibodies during ICI therapy did predict irAE development in RCC patients. However, the appearance of novel autoantibodies and irAEs during therapy may represent markers for treatment efficacy.

## Highlights

• Of 170 RCC patients treated with ICIs, 37% had pre-existing autoantibodies.
• Patients with pre-existing autoantibodies less often developed irAEs during therapy.
• 60% of baseline autoantibody-negative patients developed new autoantibodies during therapy, not linked to irAE incidence.
• The occurrence of novel autoantibodies during therapy correlated with a higher DCR for RCC patients.
• Patients with treatment-associated irAEs significantly more often achieved disease control.

## Introduction

Targeting the immune checkpoint molecules cytotoxic T-lymphocyte antigen 4, programmed cell death protein 1 (PD-1), or programmed death-ligand 1 (PD-L1) represents an attractive treatment for tumor patients.^1^^,^^2^ Immune checkpoint inhibitors (ICIs) promote T-cell-based antitumor effects, leading to objective clinical responses and improved survival in a significant number of patients with various cancers, including renal cell carcinoma (RCC).^3^ However, ICI-mediated activation of the immune system frequently causes immune-related adverse events (irAEs), which may range from mild to life-threatening^4^, ^5^, ^6^ and affect any organ, including skin, endocrine system, lung, liver, and gastrointestinal system. Interestingly, irAE occurrence may be associated with improved survival in ICI-treated tumor patients.^10^, ^7^, ^8^, ^9^

The precise immunopathogenesis driving irAE development is still unknown, but various potentially contributing mechanisms have been identified: ICIs augment proliferation, cytokine production, and cytotoxic activity of autoreactive effector T cells,^11^ and reduce blood-circulating regulatory T cells.^12^ In addition, ICI therapy can lead to a higher number of blood-circulating T helper 17 cells and increased levels of proinflammatory cytokines such as interleukin-17.^13^, ^14^, ^15^ Cross-reactivity between tumor antigens and similar antigens on healthy cells recognized by stimulated T cells may also contribute to irAE pathogenesis.^16^^,^^17^ Moreover, ICI-activated T cells can efficiently foster cytokine production, antigen-presentation capacity, and differentiation of autoreactive B cells into autoantibody-producing plasma cells.^11^ PD-1-expressing B cells can also be stimulated directly by anti-PD-1 antibodies in a T-cell-independent manner.^18^ ICI-treated cancer patients displayed a reduced number of blood-circulating B cells and increased numbers of CD21^low^ B cells and plasmablasts.^19^ These alterations were predictors of irAEs. Autoantibodies may promote irAE development by activating the complement system.^11^^,^^20^

Recent studies have explored the potential suitability of autoantibodies as predictive biomarkers for irAE development in ICI-treated tumor patients to allow an earlier management of irAEs and improve the risk–benefit balance.^21^^,^^22^ Thus, it has been demonstrated that the presence of antibodies directed against thyroid peroxidase (TPO) and thyroglobulin (TG) at baseline and the early stage of treatment may increase the risk of ICI-induced thyroid dysfunction.^23^, ^24^, ^25^, ^26^, ^27^ Similarly, patients who developed diabetes on ICI treatment displayed antibodies reactive against islet cell antigen and glutamic acid decarboxylase-65.^28^ The formation of anti-acetylcholine receptor antibodies and their association with the occurrence of myasthenia gravis following ICI therapy have also been reported.^29^ However, various studies did not find a correlation between the presence or higher levels of autoantibodies before ICI therapy and the development of irAEs.^30^, ^31^, ^32^ Based on these conflicting results and the lack of validation studies, the current guidelines do not recommend the evaluation of autoantibodies in every patient before ICI therapy.

So far, most studies investigating the potential suitability of autoantibodies as predictive biomarkers for the occurrence of ICI-mediated irAEs are retrospective and evaluate autoantibodies only before ICI initiation.^21^ To gain novel insights into an association between the presence of autoantibodies and irAE occurrence, we determined autoantibodies in a large cohort of patients with locally advanced or metastatic RCC who received a tailored approach with nivolumab ± nivolumab + ipilimumab within the multicenter, phase II clinical trial TITAN-RCC.^33^ In this prospective study, we evaluated the presence and titer of several autoantibodies that are linked to potentially occurring irAEs in sera of 170 RCC patients at baseline and at several time points during ICI treatment. We explored whether autoantibodies at baseline or newly detected titers during ICI treatment are predictive of irAE occurrence and/or associated with clinical outcome in RCC patients. We also investigated whether the presence of autoantibodies and the appearance of irAEs are associated with an improved clinical outcome of ICI-treated RCC patients.

## Patients and methods

### Study design

Within the multicenter, phase II clinical trial TITAN-RCC (NCT02917772 and EudraCT 2016-002307-26),^33^ we carried out an explorative, prospective, pre-specified analysis of sera derived from 170 ICI-treated patients with histologically confirmed locally advanced or metastatic RCC with a clear-cell component with intermediate or poor risk who were either formerly untreated (first line) or pre-treated with one prior systemic therapy (antiangiogenic or temsirolimus, second line). The clinical outcome of TITAN-RCC has been reported previously.^33^ The trial was approved by all relevant national competent authorities and independent ethics committees, and conducted in compliance with the Declaration of Helsinki,^34^ Good Clinical Practice, and local regulatory requirements. Before entering the study, all patients provided written informed consent.

The study design, including treatment regimen as well as tumor and safety assessments, has been reported previously.^33^ In brief, patients received induction nivolumab 240 mg monotherapy intravenously every 2 weeks for eight doses. Tumor assessments were carried out by computed tomography or magnetic resonance imaging at baseline, and at weeks 8 and 16 after the first dose. Based on objective response as per RECIST v1.1,^35^ (i) responders [complete/partial response (CR/PR) or suspected pseudoprogression at week 16] continued with maintenance nivolumab 240 mg monotherapy every 2 weeks until disease progression or irAE requiring discontinuation, and (ii) patients with non-response [stable/progressive disease (SD/PD) at week 16 or early PD deemed by the investigator to be clinically significant at week 8] received a boost of two doses of intravenous nivolumab (3 mg/kg body weight) and ipilimumab (1 mg/kg body weight) at 3-weekly intervals, and, if SD or PD was still present, another boost of two doses of nivolumab + ipilimumab. If responding to the first (CR/PR) or second (CR/PR/SD) two boost doses, patients were maintained with nivolumab 240 mg monotherapy every 2 weeks. (Re-)boosts were indicated for PD during nivolumab maintenance therapy in the above-mentioned schedule. Patients with PD after four consecutive nivolumab + ipilimumab boost doses were considered immunotherapy-resistant.

### Inclusion and exclusion criteria for patients

Detailed inclusion and exclusion criteria have been reported and are listed in the Supplementary Methods, available at https://doi.org/10.1016/j.esmoop.2025.105575.^33^ Known or suspected autoimmune disease or conditions requiring systemic corticosteroid treatment (>10 mg/day prednisone equivalents) were among the exclusion criteria. Adverse events were recorded from the initiation of study medication until 100 days from the last dose of study drug. They were spontaneously reported or elicited during open-ended questioning, examination, or evaluation of a subject. Adverse events were graded and coded according to the National Cancer Institute Common Terminology Criteria for Adverse Events (CTCAE) v4.0.

### Monitoring for autoantibodies

Within the TITAN-RCC trial, we prospectively analyzed anti-nuclear antibodies (ANA), anti-cytoplasmatic antibodies (ACyA), anti-mitochondrial antibodies (AMA), anti-neutrophil cytoplasmatic antibodies (ANCA), anti-TPO, anti-TG, anti-liver kidney microsome (anti-LKM) antibodies, smooth muscle antibodies (SMA), parietal cell antibodies (PCA), and anti-mitotic antibodies (AMiA) against cell cycle-dependent structures in 874 serum samples from 170 out of 207 ICI-treated RCC patients. Sera from 37 patients were not available. We determined the presence and titer of these autoantibodies in sera obtained before nivolumab induction monotherapy doses one (baseline, IP1), four (IP2), and eight (IP3), before the sixth dose of nivolumab maintenance monotherapy (MT), and before every dose with nivolumab + ipilimumab boost (BP1-4) (Supplementary Figure S1, available at https://doi.org/10.1016/j.esmoop.2025.105575).

### Measurement of autoantibody titers

Autoantibodies were explored under routine conditions and assays were carried out according to the manufacturer’s instructions. Titers were determined by serial dilution of the serum. The dilution level of the examined patient sample at which a specific fluorescence was just barely recognizable was given as the titer. Titers of immunofluorescence tests and quantitative results of enzyme-linked immunosorbent assay (ELISA) and chemoluminescence assays were appraised positive above cut-off as described below.

For determination of ANA, ACyA, and AMiA, sera were tested by indirect immunofluorescence (IIF) on HEp-2-cells (INOVA Inc., San Diego, CA). Antibody binding pattern was assessed according to the nomenclature of the International Consensus on ANA Patterns (ICAP).^36^ Titers were appraised as follows: <1 : 80 negative, 1 : 80 and 1 : 160 borderline, 1 : 320 and 1 : 640 intermediate positive, ≥1 : 1280 strong positive.

AMA, PCA, SMA, and anti-LKM were determined by IIF on rat cryostat sections of liver, kidney, and stomach (Euroimmun AG, Lübeck, Germany). Titers were appraised as follows: <1 : 10 negative, 1 : 10 borderline, 1 : 20 and 1 : 40 intermediate positive, ≥1 : 80 strong positive.

ANCA were analyzed by IIF on ethanol and formalin-fixed human neutrophils (Euroimmun AG). Patterns were distinguished into perinuclear (pANCA), cytoplasmatic (cANCA), and atypical (xANCA). Titers were appraised as follows: <1 : 10 negative, 1 : 10 borderline, 1 : 20 and 1 : 40 intermediate positive, ≥1 : 80 strong positive. In case of ANCA positivity, anti-proteinase-3 and anti-myeloperoxidase antibodies were determined by utilizing chemoluminescence assays (INOVA Inc.).

Anti-TG and anti-TPO were analyzed by ELISA (Orgentec Diagnostika GmbH, Mainz, Germany). Anti-TG results were appraised as follows: <50 IU/ml negative, ≥50-<75 IU/ml borderline, ≥75-<500 IU/ml intermediate positive, ≥500 IU/ml strong positive. Anti-TPO results were appraised as follows: <100 IU/ml negative, ≥100-<150 IU/ml borderline, ≥150-<1000 IU/ml intermediate positive, ≥1000 IU/ml strong positive.

Strong or intermediate autoantibody titers were designated as positive, whereas borderline titers or undetectable autoantibodies were designated as negative.

The assumed association between the evaluated autoantibodies and groups of irAEs is summarized in Supplementary Table S1, available at https://doi.org/10.1016/j.esmoop.2025.105575.

### Statistical analysis

Statistical calculations were carried out utilizing SPSS 27.0 (SPSS Inc., Chicago, IL). Descriptive statistics were used to create cross-tabulations. Exact chi-square test was used to analyze associations between autoantibody titers, irAEs, and treatment response in 2 × 2 cross tables. For small datasets, Fisher’s exact test was used to examine statistical significance, whereas the Cochran–Mantel–Haenszel test was applied for larger cross tables. Results were considered statistically significant at *P* < 0.05. Due to the explorative character of the investigation, no correction for multiplicity was done; however, *P* values are given for a differentiated insight.

## Results

### Detection of autoantibodies at baseline and during ICI therapy

Median patient age was 65 years. Of the patients, 71% were male and 29% were female. Approximately half of the patients were considered first line (52%) and second line (48%), respectively. Of the patients, 72% had intermediate risk, while 24% had poor risk according to the criteria introduced by the International Metastatic Renal-Cell Carcinoma Database Consortium (IMDC). Upon source data review, eight patients (5%) with favorable IMDC risk were identified. For details see Supplementary Table S2, available at https://doi.org/10.1016/j.esmoop.2025.105575.

When investigating serum autoantibody levels at baseline (IP1), autoantibodies were found in 37% (63/170) of patients. Of autoantibody-positive patients, 48% had SMA, 30% ANA, 11% PCA, 11% ACyA, 5% xANCA, 5% anti-TPO and/or anti-TG, 3% pANCA, 2% AMA, and 2% AMiA (Table 1). In 16% of patients, two or more autoantibodies with intermediate or strong positive levels were simultaneously detected at IP1 (Table 1).

**Table 1:** Autoantibody data at baseline

	Autoantibody positivity at IP1, *n* = 63 *n* (% of *n* = 63)^a^
Individual autoantibodies at IP1	63/170 (37)
SMA	30 (48)
ANA	19 (30)
PCA	7 (11)
ACyA	7 (11)
xANCA	3 (5)
Anti-TG/anti-TPO	3 (5)
pANCA	2 (3)
AMA	1 (2)
AMiA	1 (2)
Anti-LKM	0 (0)
Patients with multiple autoantibodies at IP1	10 (16)
SMA + ACyA	2 (3)
SMA + ANA	2 (3)
SMA + PCA	1 (2)
SMA + xANCA	1 (2)
ANA + anti-TG/anti-TPO	1 (2)
PCA + anti-PR3	1 (2)
AMA + ACyA	1 (2)
ANA + SMA + PCA	1 (2)

ACyA, anti-cytoplasmatic antibodies; AMA, anti-mitochondrial antibodies; AMiA, anti-mitotic antibodies; ANA, anti-nuclear antibodies; IP1, baseline time during induction phase; LKM, liver kidney microsome; pANCA, perinuclear anti-neutrophil cytoplasmatic antibodies; PCA, parietal cell antibodies; PR3, proteinase-3; SMA, smooth muscle antibodies; TG, thyroglobulin; TPO, thyroid peroxidase; xANCA, atypical anti-neutrophil cytoplasmatic antibodies.

a Unless otherwise stated.

Among the autoantibody-positive patients at baseline, a new occurrence of other autoantibody types was seen in 54% during the course of ICI therapy at various time points (Table 2). Of the patients, 22% developed new autoantibody types during induction nivolumab monotherapy before IP2 and/or IP3, 13% during nivolumab maintenance before time point MT, 13% before the first boost dose time point (BP1), and 22% after having received at least one dose of nivolumab + ipilimumab boost doses (before time points BP2-4). For details see Table 2.

**Table 2:** Autoantibody data during therapy

	Autoantibody positivity at IP1, *n* = 63 *n* (% of *n* = 63)	Autoantibody negativity at IP1, *n* = 107 *n* (% of *n* = 107)	All patients, *n* = 170 *n* (% of *n* = 170)
New occurrence of autoantibodies during therapy^a^	34 (54)	64 (60)	98 (58)
New occurrence before IP2/3	14 (22)	38 (36)	52 (31)
PCA	4 (6)	5 (5)	9 (5)
ANA	3 (5)	10 (9)	13 (8)
SMA	2 (3)	21 (20)	23 (14)
ACyA	2 (3)	3 (3)	5 (3)
xANCA	1 (2)	4 (4)	5 (3)
Anti-TG/anti-TPO	1 (2)	6 (6)	7 (4)
pANCA	1 (2)	1 (1)	2 (1)
AMiA	1 (2)	0 (0)	1 (1)
AMA	0 (0)	1 (1)	1 (1)
Anti-LKM	0 (0)	1 (1)	1 (1)
New occurrence before MT	8 (13)	11 (10)	19 (11)
PCA	7 (11)	3 (3)	10 (6)
ANA	1 (2)	5 (5)	6 (4)
SMA	1 (2)	3 (3)	4 (2)
Anti-TG/anti-TPO	1 (2)	0 (0)	1 (1)
pANCA	0 (0)	1 (1)	1 (1)
ACyA	0 (0)	0 (0)	0 (0)
xANCA	0 (0)	0 (0)	0 (0)
AMiA	0 (0)	0 (0)	0 (0)
AMA	0 (0)	0 (0)	0 (0)
Anti-LKM	0 (0)	0 (0)	0 (0)
New occurrence before BP1	8 (13)	15 (14)	23 (14)
SMA	3 (5)	10 (9)	13 (8)
PCA	4 (6)	5 (5)	9 (5)
Anti-TG/anti-TPO	2 (3)	0 (0)	2 (1)
xANCA	1 (2)	0 (0)	1 (1)
ACyA	0 (0)	0 (0)	0 (0)
Anti-LKM	0 (0)	0 (0)	0 (0)
ANA	0 (0)	1 (1)	1 (1)
pANCA	0 (0)	0 (0)	0 (0)
AMiA	0 (0)	0 (0)	0 (0)
AMA	0 (0)	0 (0)	0 (0)
New occurrence before BP2-4	14 (22)	21 (20)	35 (21)
SMA	8 (13)	12 (11)	20 (12)
PCA	5 (8)	12 (11)	17 (10)
Anti-TG/anti-TPO	0 (0)	2 (2)	2 (1)
xANCA	0 (0)	0 (0)	0 (0)
ACyA	1 (2)	1 (1)	2 (1)
Anti-LKM	1 (2)	0 (0)	1 (1)
ANA	0 (0)	1 (1)	1 (1)
pANCA	0 (0)	0 (0)	0 (0)
AMiA	0 (0)	0 (0)	0 (0)
AMA	0 (0)	0 (0)	0 (0)

ACyA, anti-cytoplasmatic antibodies; AMA, anti-mitochondrial antibodies; AMiA, anti-mitotic antibodies; ANA, anti-nuclear antibodies; BP1-4, time points 1-4 during boost phase; IP1-3, time points 1-3 during induction phase; LKM, liver kidney microsome; MT, time point during maintenance therapy; pANCA, perinuclear anti-neutrophil cytoplasmatic antibodies; PCA, parietal cell antibodies; SMA, smooth muscle antibodies; TG, thyroglobulin; TPO, thyroid peroxidase; xANCA, atypical anti-neutrophil cytoplasmatic antibodies.

a Patients could experience a new occurrence of autoantibodies during multiple study phases.

Of all patients, 63% were negative for all evaluated antibody entities at baseline. Among them, 60% developed autoantibodies during the course of ICI therapy. Of the autoantibody-negative patients at baseline, 36% showed new autoantibodies during induction nivolumab monotherapy before time points IP2 and/or IP3, 10% during maintenance therapy at time point MT, 14% before the first boost dose at time point BP1, and 20% after having received at least one dose of nivolumab + ipilimumab boost doses (before time points BP2-4). For details see Table 2.

### Detection of autoantibodies during maintenance ICI therapy

At time point MT, sera from 31% of patients were available for autoantibody detection (Supplementary Table S3, available at https://doi.org/10.1016/j.esmoop.2025.105575). Of these patients, 58% have received maintenance therapy after nivolumab induction monotherapy and 42% after nivolumab/ipilimumab boosts. Of the patients with maintenance therapy after nivolumab induction, 48% were autoantibody-positive at the time point MT (ANA *n* = 6, PCA *n* = 5, SMA *n* = 5, anti-TG/anti-TPO *n* = 2, xANCA *n* = 2). Of patients with maintenance therapy after nivolumab/ipilimumab boosts, 77% (17/22) had autoantibodies at MT.

### Correlation between pre-existing or newly occurring autoantibodies during therapy and the occurrence of irAEs

Of the ICI-treated patients, 88% developed irAEs (Table 3). Most frequent irAEs of all grades, which are commonly linked to the assessed autoantibodies, were pruritus (28%), diarrhea (24%), rash (21%), and arthralgia (10%). Most common irAEs of grade ≥3 were diarrhea (8%) and immune-mediated colitis (5%). For further details see Supplementary Table S4, available at https://doi.org/10.1016/j.esmoop.2025.105575. Thirty-four percent of patients who experienced irAEs during ICI therapy were initially autoantibody-positive, of which 19% showed a maximum CTCAE grade of 1-2 and 14% displayed irAEs of CTCAE grades 3-5. Of the 21 patients without irAEs, 62% were autoantibody-positive at IP1 (Table 3).

**Table 3:** Correlation between autoantibodies and the occurrence of immune-related adverse events

Parameter	*n*_total_ (% of *n* = 170)	irAE(−), *n* (% of *n*_total_)	irAE(+) *n* (% of *n*_total_)		*P* value
		CTCAE grade 0	CTCAE grades 1-2	CTCAE grades 3-5	
Autoantibody positivity at IP1	63 (37)	13 (21)	50 (79)		0.012^a^
Autoantibody negativity at IP1	107 (63)	8 (7)	99 (93)		
Autoantibody positivity at IP1	63 (37)	13 (21)	29 (46)	21 (33)	0.021^b^
Autoantibody negativity at IP1	107 (63)	8 (7)	48 (45)	51 (48)	
Strong autoantibody titer at IP1	35 (21)	8 (23)	27 (77)		0.001^b^
Intermediate autoantibody titer at IP1	28 (16)	5 (18)	23 (82)		
Borderline autoantibody titer at IP1	31 (18)	6 (19)	25 (81)		
Undetectable autoantibodies at IP1	76 (45)	2 (3)	74 (97)		
Strong autoantibody titer at IP1	35 (21)	8 (23)	17 (49)	10 (29)	0.004^b^
Intermediate autoantibody titer at IP1	28 (16)	5 (18)	12 (43)	11 (39)	
Borderline autoantibody titer at IP1	31 (18)	6 (19)	13 (42)	12 (39)	
Undetectable autoantibodies at IP1	76 (45)	2 (3)	35 (46)	39 (51)	
New occurrence of autoantibodies during therapy^c^					
Yes	97 (57)	8 (8)	89 (92)		0.061^a^
No	73 (43)	13 (18)	60 (82)		
Yes	97 (57)	8 (8)	45 (46)	44 (45)	0.182^b^
No	73 (43)	13 (18)	32 (44)	28 (38)	

Course of autoantibody titers during therapy.

CTCAE, Common Terminology Criteria for Adverse Events (version 4.0); IP1, baseline time during induction phase; irAE, immune-related adverse event; irAE(+), patients with at least one irAE; irAE(−), patients without irAE.

a Chi-square test.

b Cochran–Mantel–Haenszel test.

c Patients could experience a new occurrence of autoantibodies during multiple study phases.

Patients with pre-existing autoantibodies at IP1 had significantly less often irAEs during the course of therapy than autoantibody-negative patients (79% versus 93%; Table 3). While percentages of patients with irAEs of CTCAE grades 1-2 were similar for those with or without autoantibodies at IP1 (46% versus 45%), fewer patients with detectable autoantibodies at IP1 had severe irAEs of CTCAE grades 3-5 compared with autoantibody-negative patients (33% versus 48%). When exploring the different levels of autoantibody titers at IP1 separately, irAEs were reported in 77% of patients with strong titers, 82% of patients with intermediate titers, 81% of patients with borderline titers, and 97% of patients without detectable autoantibodies, again being significant. While frequencies of CTCAE grade 1-2 irAEs were similar for all initial autoantibody titers (42%-49%), patients with strong titers at IP1 were less likely to have CTCAE grade 3-5 irAEs (29%) compared with patients with intermediate titers (39%), borderline titers (39%), and undetectable autoantibodies (51%), respectively (Table 3).

There was no significant difference in frequencies or severity of irAEs between patients who experienced a new occurrence of autoantibodies during therapy and those who did not (Table 3).

### Specific autoantibodies and potentially associated irAEs

We also explored whether the various autoantibody entities at different time points are associated with the occurrence of irAEs that are commonly linked to the respective autoantibodies during the course of treatment (Supplementary Table S1, available at https://doi.org/10.1016/j.esmoop.2025.105575). We did not find a significant correlation between detectable PCA, xANCA, SMA, and ANA at baseline (IP1), before the sixth dose of maintenance nivolumab monotherapy (MT), or before nivolumab + ipilimumab boosts (BP1) with the appearance of related irAEs (Supplementary Table S5, available at https://doi.org/10.1016/j.esmoop.2025.105575). Also, while no significant association between detectable anti-TPO/anti-TG at IP1 and BP1 with the appearance of related irAEs was observed, we found a significant correlation between detectable anti-TPO/anti-TG at MT with the occurrence of thyroid dysfunction (Supplementary Table S5, available at https://doi.org/10.1016/j.esmoop.2025.105575).

### Correlation between autoantibodies and response to ICI treatment

The objective response rate for nivolumab ± nivolumab + ipilimumab was 35% for all patients assessed, with a complete response rate of 7% (Supplementary Table S6, available at https://doi.org/10.1016/j.esmoop.2025.105575).

Of the patients with intermediate or strong autoantibody titers at IP1, 52% achieved disease control (CR/PR/SD) as best overall response to nivolumab ± nivolumab + ipilimumab boosts (Table 4). Numerically, the disease control rate (DCR) was higher for autoantibody-positive patients at IP1 (65%), however, without significance. When considering the different levels of autoantibody titers at IP1 separately, DCR was not linked to autoantibody titers.

**Table 4:** Correlation between autoantibodies and response to ICI therapy

Parameter		*n*_total_ (% of *n* = 170)	BOR, *n* (% of *n*_total_)		*P* value
			CR/PR/SD	PD	
Autoantibody positivity at IP1		63 (37)	33 (52)	30 (48)	0.093^a^
Autoantibody negativity at IP1		107 (63)	70 (65)	37 (35)	
Strong autoantibody titer at IP1		35 (21)	18 (51)	17 (49)	0.175^b^
Intermediate autoantibody titer at IP1		28 (16)	15 (54)	13 (46)	
Borderline autoantibody titer at IP1		31 (18)	22 (71)	9 (29)	
Undetectable autoantibodies at IP1		76 (45)	48 (63)	28 (37)	
New occurrence of any autoantibody during therapy^c^					
Any autoantibody positivity	Yes	97 (57)	73 (75)	24 (25)	<0.001^a^
	No	73 (43)	30 (41)	43 (59)	
SMA positivity	Yes	60 (35)	48 (80)	12 (20)	<0.001^a^
	No	110 (65)	55 (50)	55 (50)	
ANA positivity	Yes	22 (13)	19 (86)	3 (14)	0.008^a^
	No	148 (87)	84 (57)	64 (43)	
xANCA positivity	Yes	7 (4)	7 (100)	0 (0)	0.029^a^
	No	163 (96)	96 (59)	67 (41)	
PCA positivity	Yes	46 (27)	33 (72)	13 (28)	0.070^a^
	No	124 (73)	70 (56.5)	54 (43.5)	
Anti-TG/anti-TPO positivity	Yes	12 (7)	10 (83)	2 (17)	0.094^a^
	No	158 (93)	93 (59)	65 (41)	
ACyA positivity	Yes	7 (4)	2 (29)	5 (71)	0.077^a^
	No	163 (96)	101 (62)	62 (38)	
pANCA positivity	Yes	3 (2)	3 (100)	0 (0)	0.159^a^
	No	167 (98)	100 (60)	67 (40)	
Anti-LKM positivity	Yes	2 (1)	1 (50)	1 (50)	0.758^a^
	No	168 (99)	102 (61)	66 (39)	
AMA positivity	Yes	1 (0.6)	1 (100)	0 (0)	0.419^a^
	No	169 (99.4)	102 (60)	67 (40)	
AMiA positivity	Yes	1 (0.6)	0 (0)	1 (100)	0.214^a^
	No	169 (99.4)	103 (61)	66 (39)	

Course of autoantibody titers during therapy. Patients could experience increase in multiple autoantibody titers.

ACyA, anti-cytoplasmatic antibodies; AMA, anti-mitochondrial antibodies; AMiA, anti-mitotic antibodies; ANA, anti-nuclear antibodies; BOR, best overall response; CR, complete response; IP1, baseline time during induction phase; LKM, liver kidney microsome; pANCA, perinuclear anti-neutrophil cytoplasmatic antibodies; PCA, parietal cell antibodies; PD, progressive disease; PR, partial response; SD, stable disease; SMA, smooth muscle antibodies; TG, thyroglobulin; TPO, thyroid peroxidase; xANCA, atypical anti-neutrophil cytoplasmatic antibodies.

a Chi-square test.

b Cochran–Mantel–Haenszel test.

c Patients could experience a new occurrence of autoantibodies during multiple study phases.

Of the patients, 57% newly developed any autoantibodies during therapy (i.e. from initial undetectable autoantibodies or borderline titers to intermediate or strong titers). Among them, 75% achieved disease control compared with 41% in patients without new occurrence of autoantibodies during therapy. Regarding the different autoantibody entities, 80% of patients who developed SMA during therapy achieved DCR. This applied to 50% of patients who did not develop SMA during therapy. A significant correlation was also seen between patients with/without a novel occurrence of ANA or xANCA and treatment response (Table 4). There was no significant correlation between response (disease control versus PD) and new occurrence of PCA, anti-TG/anti-TPO, ACyA, pANCA, anti-LKM, AMA, or AMiA.

### Correlation between irAEs and response to ICI treatment

When correlating disease control and occurrence of irAEs, DCR was higher if patients experienced at least one irAE [irAE(+) 67% versus irAE(−) 14%; Table 5]. Considering the highest CTCAE grade of irAEs that occurred per patient, DCRs of subjects with CTCAE grades 1-2 and grades 3-5 were comparable (66% versus 68%). When exploring irAEs associated with specific autoantibodies, DCR was significantly higher in patients who developed pruritus versus those who did not (80% versus 53%). This also applied to patients who developed cutaneous irAEs (rash, erythema, skin exfoliation, blister, dermatitis) versus those who did not (77% versus 53%), to patients who showed either arthralgia, myalgia, or musculoskeletal pain versus those who did not (81% versus 55%), as well as to patients who developed hypothyroidism, hyperthyroidism, or thyroid-stimulating hormone increased/decreased versus those who did not (78% versus 57%). For all other irAEs associated with particular autoantibodies, no correlation was found between the occurrence of the respective irAE and the response (disease control versus PD) to ICI therapy (Table 5).

**Table 5:** Correlation between response and immune-related adverse events

Parameter		*n*_total_ (% of *n* = 170)	BOR, *n* (% of *n*_total_)		*P* value
			CR/PR/SD	PD	
irAEs(–)		21 (12)	3 (14)	18 (86)	<0.001^a^
irAEs(+)		149 (88)	100 (67)	49 (33)	
irAE CTCAE grade 0		21 (12)	3 (14)	18 (86)	0.005^b^
irAE CTCAE grades 1-2		77 (45)	51 (66)	26 (34)	
irAE CTCAE grades 3-5		72 (42)	49 (68)	23 (32)	
ANA-associated irAEs					
Pruritus	Yes	50 (29)	40 (80)	10 (20)	0.001^a^
	No	120 (71)	63 (53)	57 (47.5)	
Cutaneous adverse events^c^	Yes	56 (33)	43 (77)	13 (23)	0.002^a^
	No	114 (67)	60 (53)	54 (47)	
Arthralgia, myalgia, musculoskeletal pain	Yes	36 (21)	29 (81)	7 (19)	0.006^a^
	No	134 (79)	74 (55)	60 (45)	
Peripheral facial paresis, paraparesis	Yes	3 (2)	2 (67)	1 (33)	0.828^a^
	No	167 (98)	101 (60.5)	66 (39.5)	
Polyarthritis	Yes	4 (2)	3 (75)	1 (25)	0.551^a^
	No	166 (98)	100 (60)	66 (40)	
PCA-associated irAEs					
Gastritis, stomatitis mucosal inflammation, gastroenteritis	Yes	10 (6)	6 (60)	4 (40)	0.969^a^
	No	160 (94)	97 (61)	63 (39)	
SMA-associated irAEs					
Hepatotoxicity, immune-mediated hepatitis	Yes	5 (3)	4 (80)	1 (20)	0.367^a^
	No	165 (97)	99 (60)	66 (40)	
Liver value increased (alanine/aspartate aminotransferases)	Yes	22 (13)	16 (73)	6 (27)	0.212^a^
	No	148 (87)	87 (59)	61 (41)	
xANCA-associated irAEs					
Colitis, diverticulitis, immune-mediated enterocolitis	Yes	16 (9)	10 (62.5)	6 (37.5)	0.869^a^
	No	154 (91)	93 (60)	61 (40)	
Diarrhea, constipation	Yes	43 (25)	31 (72)	12 (28)	0.074^a^
	No	127 (75)	72 (57)	55 (43)	
Pancreatitis and/or autoimmune pancreatitis	Yes	3 (2)	2 (67)	1 (33)	0.828^a^
	No	167 (98)	101 (60.5)	66 (39.5)	
Anti-TG/anti-TPO-associated irAEs					
Hypothyroidism, hyperthyroidism, thyroid-stimulating hormone increased/decreased	Yes	27 (16)	21 (78)	6 (22)	0.046^a^
	No	143 (84)	82 (57)	61 (43)	

ANA, anti-nuclear antibodies; BOR, best overall response; CR, complete response; CTCAE, Common Terminology Criteria for Adverse Events (version 4.0); irAE, immune-related adverse event; PCA, parietal cell antibodies; PD, progressive disease; PR, partial response; SD, stable disease; SMA, smooth muscle antibodies; TG, thyroglobulin; TPO, thyroid peroxidase; xANCA, atypical anti-neutrophil cytoplasmatic antibodies.

a Chi-square test.

b Cochran–Mantel–Haenszel test.

c Cutaneous adverse events: rash, erythema, skin exfoliation, blister, dermatitis.

## Discussion

In our prospective study, we evaluated the presence of various autoantibody entities that are linked to common irAEs in sera of 170 RCC patients at baseline and at several time points during nivolumab maintenance monotherapy and nivolumab + ipilimumab boost within the TITAN-RCC multicenter trial.^33^ In 37% of patients, we found autoantibodies at baseline, with SMA, ANA, and PCA being most frequent. A new occurrence of autoantibody entities was seen in 54% and 60% of patients with and without pre-existing autoantibodies, respectively. Of all patients, 88% developed irAEs of any grade during ICI therapy, with pruritus, diarrhea, rash, and arthralgia being most frequent. RCC patients with pre-existing autoantibodies significantly less often developed irAEs during therapy. Interestingly, this difference was attributable to severe irAEs of grades 3-5. A new occurrence of the majority of autoantibodies during ICI treatment was not significantly associated with the incidence of irAEs in RCC patients. However, we found a significant correlation between the presence of anti-TPO/anti-TG at MT with the development of thyroid dysfunction.

To date, most studies exploring the suitability of autoantibodies as predictive biomarkers for irAE occurrence are retrospective, include a small cohort of patients, and determine autoantibodies only at baseline.^21^ In a retrospective study involving 83 ICI-treated non-small-cell lung cancer (NSCLC) patients, 21.7% were ANA-positive before therapy. ANA positivity was not significantly associated with a higher irAE incidence.^37^ Mouri et al. reported that 44.7% of 266 ICI-treated NSCLC patients were ANA-positive at baseline. No significant differences in the incidence of irAEs between ANA-positive and -negative patients were found.^30^ Alserawan et al. also found no association between ANA positivity and irAE occurrence, when exploring 134 ICI-treated patients with different cancer entities.^31^ However, 22 patients developed new ANA patterns after ICI initiation, which were linked to higher rates of severe irAEs than in patients with ANA positivity at baseline. Another study analyzed a broad spectrum of autoantibodies in sera from 133 melanoma patients collected before and 12 weeks after ICI treatment.^38^ Again, no significant correlation between pre-existing autoantibodies and irAE development was observed. Autoantibodies developed in 19.2% of patients who were autoantibody-negative before treatment, and the development of any autoantibodies and any irAEs was not significantly correlated. In a retrospective study comprising 275 ICI-treated patients with various cancers, no significant association was found between the presence of autoantibodies before therapy and irAE occurrence.^39^ However, thyroid dysfunction was observed more frequently in patients with pre-existing anti-TG or anti-TPO. Carrying out a prospective pan-cancer study, Barth et al. showed that the presence of autoantibodies at baseline was not associated with the frequency of irAEs in 44 ICI-treated patients.^40^ Furthermore, Ghosh et al. examined a broad spectrum of autoantibodies in 60 melanoma patients before and 6 weeks after ICI treatment, and demonstrated that patients with high baseline autoantibody levels were less likely to experience various irAEs.^32^ However, patients with a greater fold change in autoantibody concentration from baseline to 6 weeks developed more organ-specific irAEs.^32^ A significant association between organ-specific irAEs and autoantibodies associated with autoimmune diseases in those targeted organs was not observed. In line with this study, we found that patients with pre-existing autoantibodies significantly less often developed irAEs during ICI therapy and that patients with strong autoantibody titers at baseline were less likely to experience CTCAE grade 3-5 irAEs. The underlying mechanisms for this finding are unclear. However, various factors such as pre-existing autoimmune diseases, pre-treatment, and gender can influence the presence of autoantibodies at baseline. We did not include patients with autoimmune diseases at baseline in our study. In addition, pre-treatment with tyrosine kinase inhibitors or gender was not significantly associated with the presence of pre-existing autoantibodies in patients.

In contrast, other studies showed a correlation between autoantibody presence at baseline and irAE incidence in ICI-treated cancer patients. Toi et al. retrospectively determined autoantibodies in 137 ICI-treated NSCLC patients and demonstrated that pre-existing autoantibodies are significantly and independently associated with irAE development.^41^ Furthermore, a retrospective study of 92 ICI-treated NSCLC patients showed that early serum conversion of autoantibodies (within 30 days of treatment initiation) was significantly correlated with irAE incidence.^42^ Genta et al. retrospectively analyzed autoantibodies in 114 ICI-treated patients with different cancer entities before and after ICI treatment.^43^ They observed that patients who developed grade ≥2 irAEs had a higher number of autoantibody reactivities at baseline than those without irAEs, and an increase in post-treatment immunoglobulin M reactivities in patients experiencing two or more irAEs. In another retrospective study, ANA positivity was reported in 9 of 191 patients and was significantly associated with colitis development.^44^ Another retrospective study of 68 ICI-treated urothelial carcinoma patients showed that 30% of patients had an ANA titer of >1/160 at baseline, which was significantly associated with a higher incidence of irAEs.^45^ Furthermore, a retrospective study of 159 ICI-treated NSCLC patients revealed that an ANA titer of ≥1/320 at baseline was linked to irAEs and a higher incidence of skin adverse reactions.^46^ In a prospective pan-cancer study of 221 ICI-treated patients, pre-existing autoantibodies were found in 58.4% of patients.^47^ Grade ≥2 irAEs were more frequent and occurred earlier in patients with pre-existing autoantibodies.

Recent studies have also investigated a potential association between the presence of autoantibodies at baseline or during ICI therapy and clinical outcome, with conflicting results. Thus, pre-existing autoantibodies and an early serum conversion (within 30 days since treatment initiation) of autoantibodies were significantly associated with improved clinical outcome in ICI-treated NSCLC patients.^41^^,^^42^ Occurrence of irAEs was linked to prolonged overall survival (OS) and progression-free survival (PFS). In contrast, Yoneshima et al. found that survival was significantly shorter in ANA-positive NSCLC patients treated with ICI than in ANA-negative patients.^37^ Two other retrospective studies found no correlation between ANA titers >1/160 or ≥1/320 and survival in patients with urothelial carcinoma or NSCLC.^45^^,^^46^ In addition, two prospective, pan-cancer studies comprising 44 or 221 ICI-treated patients reported that pre-existing autoantibodies were not correlated with clinical outcome.^40^^,^^47^ However, PFS and OS were significantly longer in patients with grade ≥2 irAEs compared with those without irAEs. Furthermore, de Moel et al. reported that melanoma patients who developed autoantibodies showed a trend for better survival, whereas a link between irAE occurrence and clinical outcome was not observed.^38^ In our prospective study as part of the TITAN-RCC trial,^33^ we did not observe a significant association between pre-existing autoantibodies and achieved disease control. However, we found a significant correlation between the occurrence of newly developed autoantibodies or irAEs during treatment and a higher DCR in ICI-treated RCC patients.

Strengths of our study comprise the prospective design, the large cohort of ICI-treated RCC patients, and the assessment of autoantibodies at various time points during therapy. However, our study also has limitations. Thus, due to the tailored study design, a significant proportion of RCC patients did not receive nivolumab maintenance therapy or boosts, leading to a reduced number of available serum samples at MT or BP1-4 compared with baseline. Additionally, our findings have to be validated in another prospective, multicenter clinical trial.

## Conclusions

Previous studies demonstrated conflicting results regarding an association between the presence of autoantibodies at baseline or during ICI therapy and irAE development or clinical outcome in cancer patients. Thus, we and others provided evidence that the presence of pre-existing autoantibodies or the occurrence of the majority of analyzed autoantibodies during ICI treatment neither predicts the appearance of irAEs nor contradicts ICI therapy. Further results revealed that the presence of autoantibodies at baseline or the appearance of new autoantibodies during ICI therapy is associated with a better clinical outcome and may represent a marker of treatment efficacy. In contrast, other studies showed an association between pre-existing autoantibodies and irAE incidence, but not between autoantibodies at baseline and improved clinical outcome in ICI-treated cancer patients. Differences in the tumor type, pre-treatment, ICI therapy regimen, gender, as well as genetic and environmental factors of the patient cohort may contribute to the conflicting findings. Therefore, further comprehensive and prospective multicenter trials are required, taking these potentially influencing factors into account. Further studies should also explore B cells and T lymphocytes that are essential for the presence of pre-existing autoantibodies or the occurrence of novel autoantibodies during ICI therapy.

## References

[1] Ribas A., Wolchok J.D. (2018). Cancer immunotherapy using checkpoint blockade. Science.

[2] Sharma P., Goswami S., Raychaudhuri D. (2023). Immune checkpoint therapy—current perspectives and future directions. Cell.

[3] Topalian S.L., Hodi F.S., Brahmer J.R. (2019). Five-year survival and correlates among patients with advanced melanoma, renal cell carcinoma, or non–small cell lung cancer treated with nivolumab. JAMA Oncol.

[4] Postow M.A., Sidlow R., Hellmann M.D. (2018). Immune-related adverse events associated with immune checkpoint blockade. N Engl J Med.

[5] Johnson D.B., Nebhan C.A., Moslehi J.J., Balko J.M. (2022). Immune-checkpoint inhibitors: long-term implications of toxicity. Nat Rev Clin Oncol.

[6] Iwama S., Kobayashi T., Arima H. (2025). Management, biomarkers and prognosis in people developing endocrinopathies associated with immune checkpoint inhibitors. Nat Rev Endocrinol.

[7] Eggermont A.M., Kicinski M., Blank C.U. (2020). Association between immune-related adverse events and recurrence-free survival among patients with stage III melanoma randomized to receive pembrolizumab or placebo: a secondary analysis of a randomized clinical trial. JAMA Oncol.

[8] Park R., Lopes L., Saeed A. (2021). Anti-PD-1/L1-associated immune-related adverse events as harbinger of favorable clinical outcome: systematic review and meta-analysis. Clin Transl Oncol.

[9] Kawai T., Taguchi S., Nakagawa T. (2022). Impact of immune-related adverse events on the therapeutic efficacy of pembrolizumab in urothelial carcinoma: a multicenter retrospective study using time-dependent analysis. J Immunother Cancer.

[10] Blum S.M., Rouhani S.J., Sullivan R.J. (2023). Effects of Immune-related adverse events (irAEs) and their treatment on antitumor immune responses. Immunol Rev.

[11] Ramos-Casals M., Brahmer J.R., Callahan M.K. (2020). Immune-related adverse events of checkpoint inhibitors. Nat Rev Dis Primers.

[12] de Coaña Y.P., Poschke I., Gentilcore G. (2013). Ipilimumab treatment results in an early decrease in the frequency of circulating granulocytic myeloid-derived suppressor cells as well as their Arginase1 production. Cancer Immunol Res.

[13] von Euw E., Chodon T., Attar N. (2009). CTLA4 blockade increases Th17 cells in patients with metastatic melanoma. J Transl Med.

[14] Tarhini A.A., Zahoor H., Lin Y. (2015). Baseline circulating IL-17 predicts toxicity while TGF-β1 and IL-10 are prognostic of relapse in ipilimumab neoadjuvant therapy of melanoma. J Immunother Cancer.

[15] Knochelmann H.M., Dwyer C.J., Bailey S.R. (2018). When worlds collide: Th17 and Treg cells in cancer and autoimmunity. Cell Mol Immunol.

[16] Wucherpfennig K.W., Sethi D. (2011). T cell receptor recognition of self and foreign antigens in the induction of autoimmunity. Semin Immunol.

[17] Berner F., Bomze D., Diem S. (2019). Association of checkpoint inhibitor–induced toxic effects with shared cancer and tissue antigens in non–small cell lung cancer. JAMA Oncol.

[18] Thibult M.L., Mamessier E., Gertner-Dardenne J. (2013). PD-1 is a novel regulator of human B-cell activation. Int Immunol.

[19] Das R., Bar N., Ferreira M. (2018). Early B cell changes predict autoimmunity following combination immune checkpoint blockade. J Clin Invest.

[20] Singh N., Hocking A.M., Buckner J.H. (2023). Immune-related adverse events after immune check point inhibitors: understanding the intersection with autoimmunity. Immunol Rev.

[21] Les I., Martínez M., Pérez-Francisco I. (2023). Predictive biomarkers for checkpoint inhibitor immune-related adverse events. Cancers.

[22] Taylor J., Gandhi A., Gray E., Zaenker P. (2023). Checkpoint inhibitor immune-related adverse events: a focused review on autoantibodies and B cells as biomarkers, advancements and future possibilities. Front Immunol.

[23] Osorio J., Ni A., Chaft J. (2017). Antibody-mediated thyroid dysfunction during T-cell checkpoint blockade in patients with non-small-cell lung cancer. Ann Oncol.

[24] Kimbara S., Fujiwara Y., Iwama S. (2018). Association of antithyroglobulin antibodies with the development of thyroid dysfunction induced by nivolumab. Cancer Sci.

[25] Kobayashi T., Iwama S., Yasuda Y. (2018). Patients with antithyroid antibodies are prone to develop destructive thyroiditis by nivolumab: a prospective study. J Endocr Soc.

[26] Borgers J.S., van Wesemael T.J., Gelderman K.A. (2024). Autoantibody-positivity before and seroconversion during treatment with anti-PD-1 is associated with immune-related adverse events in patients with melanoma. J Immunother Cancer.

[27] Liu J., Chen M., Li S. (2024). Biomarkers in the early stage of PD-1 inhibitor treatment have shown superior predictive capabilities for immune-related thyroid dysfunction. Front Immunol.

[28] Stamatouli A.M., Quandt Z., Perdigoto A.L. (2018). Collateral damage: insulin-dependent diabetes induced with checkpoint inhibitors. Diabetes.

[29] Johansen A., Christensen S.J., Scheie D., Højgaard J.L., Kondziella D. (2019). Neuromuscular adverse events associated with anti-PD-1 monoclonal antibodies: systematic review. Neurology.

[30] Mouri A., Kaira K., Yamaguchi O. (2021). Efficacy and feasibility of programmed death-1/programmed death ligand-1 blockade therapy in non-small cell lung cancer patients with high antinuclear antibody titers. Front Oncol.

[31] Alserawan L., Anguera G., Zamora Atenza C. (2022). Association between changes in the patterns of antinuclear autoantibodies during immune checkpoint inhibition therapy and the development of severe immune related adverse events. Int J Mol Sci.

[32] Ghosh N., Postow M., Zhu C. (2022). Lower baseline autoantibody levels are associated with immune-related adverse events from immune checkpoint inhibition. J Immunother Cancer.

[33] Grimm M.O., Esteban E., Barthélémy P. (2023). Tailored immunotherapy approach with nivolumab with or without nivolumab plus ipilimumab as immunotherapeutic boost in patients with metastatic renal cell carcinoma (TITAN-RCC): a multicentre, single-arm, phase 2 trial. Lancet Oncol.

[34] World Medical Association Declaration of Helsinki (1997). Recommendations guiding physicians in biomedical research involving human subjects. J Am Med Assoc.

[35] Eisenhauer E.A., Therasse P., Bogaerts J. (2009). New response evaluation criteria in solid tumours: revised RECIST guideline (version 1.1). Eur J Cancer.

[36] Chan E.K., von Mühlen C.A., Fritzler M.J. (2022). The international consensus on ANA patterns (ICAP) in 2021—the 6th workshop and current perspectives. J Appl Lab Med.

[37] Yoneshima Y., Tanaka K., Shiraishi Y. (2019). Safety and efficacy of PD-1 inhibitors in non–small cell lung cancer patients positive for antinuclear antibodies. Lung Cancer.

[38] de Moel E.C., Rozeman E.A., Kapiteijn E.H. (2019). Autoantibody development under treatment with immune-checkpoint inhibitors. Cancer Immunol Res.

[39] Izawa N., Shiokawa H., Onuki R. (2022). The clinical utility of comprehensive measurement of autoimmune disease-related antibodies in patients with advanced solid tumors receiving immune checkpoint inhibitors: a retrospective study. ESMO Open.

[40] Barth D.A., Stanzer S., Spiegelberg J. (2022). Evaluation of autoantibodies as predictors of treatment response and immune-related adverse events during the treatment with immune checkpoint inhibitors: a prospective longitudinal pan-cancer study. Cancer Med.

[41] Toi Y., Sugawara S., Sugisaka J. (2019). Profiling preexisting antibodies in patients treated with anti–PD-1 therapy for advanced non–small cell lung cancer. JAMA Oncol.

[42] Giannicola R., D’Arrigo G., Botta C. (2019). Early blood rise in auto-antibodies to nuclear and smooth muscle antigens is predictive of prolonged survival and autoimmunity in metastatic-non-small cell lung cancer patients treated with PD-1 immune-check point blockade by nivolumab. Mol Clin Oncol.

[43] Genta S., Lajkosz K., Yee N.R. (2023). Autoimmune paneLs as prEdictors of toxicity in patients TReated with immune checkpoint inhibiTors (ALERT). J Exp Clin Cancer Res.

[44] Sakakida T., Ishikawa T., Chihara Y. (2020). Safety and efficacy of PD-1/PD-L1 blockade in patients with preexisting antinuclear antibodies. Clin Transl Oncol.

[45] Castel-Ajgal Z., Goulvestre C., Zaibet S. (2022). Preexisting autoantibodies and immune related adverse events in metastatic urothelial carcinoma patients treated by pembrolizumab. Clin Genitourin Cancer.

[46] Zhang D., Shi Y., Liu X. (2022). Safety and efficacy of immune checkpoint inhibitors in non-small cell lung cancer patients with preexisting antinuclear antibodies: a retrospective cohort study. Transl Lung Cancer Res.

[47] Daban A., Gonnin C., Phan L. (2023). Preexisting autoantibodies as predictor of immune related adverse events (irAEs) for advanced solid tumors treated with immune checkpoint inhibitors (ICIs). OncoImmunology.

