# Supplementary material for: Profiling autoantibodies at baseline and during immune checkpoint inhibitor therapy of renal cell carcinoma patients—exploratory results from TITAN-RCC
Source: ESMO Open. 2025 Aug 28;10(9):105575. doi: 10.1016/j.esmoop.2025.105575 (PMC12414892; doi:10.1016/j.esmoop.2025.105575)
Supplement: Supplementary Material [file mmc1.docx]

**Supplementary Materials**

**Supplementary Methods – Inclusion and exclusion criteria**

**Key inclusion criteria**

- - Subjects or legally acceptable representatives must have signed written informed consent form in accordance with regulatory and institutional guidelines
  - Males and females, ≥18 years of age
  - Histological confirmation of renal cell carcinoma (RCC) with a clear-cell component
  - Advanced (not amenable to curative surgery or radiation therapy) or metastatic RCC
  - One (antioangiogenic or temsirolimus) or no prior systemic therapy for RCC with the following exception: One prior adjuvant or neoadjuvant therapy for completely resectable RCC if such therapy did not include an agent that targets VEGF or VEGF receptors as well as CTLA-4- or PD-1/PD-L1 immune checkpoint inhibitors, respectively, and if recurrence occurred at least 6 months after the last dose of adjuvant or neoadjuvant therapy.
  - Karnofsky Performance Status (KPS) of at least 70%
  - Measurable disease as per RECIST v1.1
  - Tumor tissue (formalin-fixed paraffin-embedded archival or recent acquisition) must be received by the central pathology (block or unstained slides). (Note: fine needle aspiration and bone metastases samples are not acceptable for submission).
  - Risk categories intermediate and poor (according to the International Metastatic RCC Database Consortium, IMDC):

*To be eligible as risk group intermediate or poor, at least one of the following prognostic factors as per the International Metastatic RCC Database Consortium (IMDC) criteria must be present:*

- 1. *KPS equal to 70%*
  2. *Less than 1 year from initial diagnosis of RCC (eg, nephrectomy or first diagnostic biopsy) to registration (first-line) or to start of first-line targeted therapy (second-line), respectively*
  3. *Hemoglobin less than the lower limit of normal (LLN)*
  4. *Corrected calcium concentration greater than the upper limit of normal (ULN)*
  5. *Absolute neutrophil count greater than the ULN*
  6. *Platelet count greater than the ULN*

*If none of the above factors are present, subjects are not eligible (risk group favorable).*

**Key exclusion criteria**

***Target Disease Exceptions***

- - any history of or current CNS metastases (baseline imaging of the brain by MRI (preferred) or CT scan is required within 28 days prior to registration)

***Medical History and Concurrent Diseases***

- - Prior systemic treatment with more than one of the following drugs: mTOR, VEGF or VEGF receptor targeted therapy (including, but not limited to, temsirolimus, everolinus, sunitinib, pazopanib, axitinib, tivozanib, and bevacizumab).
  - Prior treatment with an anti-PD-1, anti-PD-L1, anti-PD-L2, anti-CD137, or anti‑CTLA‑4 antibody, or any other antibody or drug specifically targeting T-cell co-stimulation or checkpoint pathways.
  - Any active or recent history of a known or suspected autoimmune disease or recent history of a syndrome that required systemic corticosteroids (> 10 mg daily prednisone equivalent) or immunosuppressive medications except for syndromes which would not be expected to recur in the absence of an external trigger. Subjects with vitiligo or type I diabetes mellitus or residual hypothyroidism due to autoimmune thyroiditis only requiring hormone replacement are permitted to enroll.
  - Any condition requiring systemic treatment with corticosteroids (> 10 mg daily prednisone equivalents) or other immunosuppressive medications within 14 days prior to first dose of study drug. Inhaled steroids and adrenal replacement steroid doses > 10 mg daily prednisone equivalents are permitted in the absence of active autoimmune disease.
  - Prior malignancy active within the previous 3 years except for locally curable cancers that have been apparently cured, such as basal or squamous cell skin cancer, superficial bladder cancer, or carcinoma in situ of the prostate, cervix, or breast.
  - Human immunodeficiency virus (HIV) infection or known acquired immunodeficiency syndrome (AIDS).
  - Any positive test for hepatitis B or hepatitis C virus indicating acute or chronic infection.
  - Known medical condition (eg, a condition associated with diarrhea or acute diverticulitis) that, in the investigator’s opinion, would increase the risk associated with study participation or study drug administration or interfere with the interpretation of safety results.
  - Major surgery (eg, nephrectomy) less than 28 days prior to the first dose of study drug.
  - Anti-cancer therapy less than 28 days prior to the first dose of study drug or palliative, focal radiation therapy less than 14 days prior to the first dose of study drug.
  - Presence of any toxicities attributed to prior anti-cancer therapy, other than alopecia, that have not resolved to Grade 1 (NCI CTCAE v4) or baseline before administration of study drug.

***Any of the following laboratory test findings***

- - White blood cell count <2,000/mm^3^
  - Neutrophils <1,500/mm^3^
  - Platelets <100,000/mm^3^
  - Alanine or aspartate transaminases >3xULN (>5xULN if liver metastases are present)
  - Total Bilirubin >1.5xULN (except subjects with Gilbert Syndrome, who can have total bilirubin <3.0 mg/dL)
  - Serum creatinine >1.5xULN or creatinine clearance <40mL/min (measured or calculated by Cockroft-Gault formula)

**Supplementary Table S-1. Assumed association between specific autoantibodies and groups of immune-related adverse events.**

| **Autoantibody** | **Groups of associated immune-related adverse events** |
| --- | --- |
| **ANA** | Skin affections: pruritus, rash, erythema, skin exfoliation, blister, dermatitis  Neural affections: peripheral facial paresis, paraparesis  Musculoskeletal involvement: arthralgia, myalgia, musculoskeletal pain, polyarthritis |
| **pANCA / cANCA** | Vasculitis |
| **xANCA** | Gastrointestinal affections: colitis, diverticulitis, immune-mediated enterocolitis, diarrhea, constipation, pancreatitis, autoimmune pancreatitis |
| **PCA** | Gastric involvement: gastritis, stomatitis, mucosal inflammation, gastroenteritis |
| **SMA** | Liver involvement: hepatotoxicity, immune mediated hepatitis, liver value increases (alanine / aspartate aminotransferases) |
| **Anti-TPO/anti-TG** | Thyroid involvement: hypothyroidism, hyperthyroidism, thyroid-stimulating hormone increase/decrease |

**Supplementary Table S-2.** **Baseline characteristics.**

^a)^ In France, recording of race is unlawful.

IMDC, International Metastatic Renal Cell Carcinoma Database Consortium; IQR, interquartile range; LLN, lower limit of normal; ULN, upper limit of normal.

|  | **All patients** n=170 |
| --- | --- |
| **Median age, years (IQR)** | 65 (56–71) |
| **Sex, n (%)** |  |
| Female | 49 (29) |
| Male | 121 (71) |
| **Race, n (%)** |  |
| White | 123 (72) |
| Black or African American | 1 (0.6) |
| Missing / not reported^a)^ | 46 (27) |
| **IMDC risk factors, n (%)** |  |
| Karnofsky Performance Status <80% | 24 (14) |
| Initial RCC diagnosis <12 months | 110 (65) |
| Haemoglobin <LLN | 90 (53) |
| Platelet count >ULN | 33 (19) |
| Neutrophil count (absolute%) >ULN | 19 (11) |
| Calcium (corrected%) >ULN | 24 (14) |
| **IMDC risk group, n (%)** |  |
| Favorable | 8 (5) |
| Intermediate | 122 (72) |
| Poor | 40 (24) |
| **Line of treatment, n (%)** |  |
| First-line | 88 (52) |
| Second-line | 82 (48) |

**Supplementary Table S-3. Autoantibody data during maintenance therapy.**

From 53 patients data from time point MT during maintenance therapy were available. ^a)^ Patients could experience autoantibody-positivity in multiple autoantibodies.

MT, time point during maintenance therapy

|  | **Autoantibody-positivity at MT after induction phase,  n=31** (% of n=31) | **Autoantibody-positivity at MT after boost phase,  n=22** (% of n=22) |
| --- | --- | --- |
| **Any autoantibody-positivity at MT** | **15 (48)** | **17 (77)** |
| ANA | 6 (19) | 5 (23) |
| PCA | 5 (16) | 10 (45) |
| SMA | 5 (16) | 7 (32) |
| Anti-TG/anti-TPO | 2 (6) | 2 (9) |
| xANCA | 2 (6) | 1 (5) |

**Supplementary Table S-4.** **Treatment-related adverse events.**

Reported treatment-related adverse events comprise all that were associated to the assessed autoantibodies. Patients could experience several treatment-related adverse events. Reported as preferred MedDRA (Medical Dictionary for Regulatory Activities) terms.

| **n (%)** | **All patients** n=170 | |
| --- | --- | --- |
|  | **All grades** | **Grades 3-5** |
| Pruritus | 47 (28) | 0 (0) |
| Diarrhea | 41 (24) | 13 (8) |
| Rash | 35 (21) | 2 (1) |
| Arthralgia | 17 (10) | 2 (1) |
| Hypothyroidism | 14 (8) | 1 (0.6) |
| Hyperthyroidism | 13 (8) | 0 (0) |
| Musculoskeletal pain | 12 (7) | 0 (0) |
| Alanine aminotransferase increased | 10 (6) | 2 (1) |
| Immune-mediated enterocolitis | 9 (5) | 8 (5) |
| Myalgia | 9 (5) | 0 (0) |
| Aspartate aminotransferase increased | 8 (5) | 0 (0) |
| Erythema | 8 (5) | 0 (0) |
| Colitis | 6 (4) | 3 (2) |
| Mucosal inflammation | 6 (4) | 1 (0.6) |
| Stomatitis | 4 (2) | 1 (0.6) |
| Pruritus generalized | 3 (2) | 0 (0) |
| Thyroid-stimulating hormone decreased | 3 (2) | 0 (0) |
| Thyroid-stimulating hormone increased | 3 (2) | 0 (0) |
| Constipation | 2 (1) | 0 (0) |
| Dermatitis exfoliative generalised | 2 (1) | 1 (0.6) |
| Hepatotoxicity | 2 (1) | 0 (0) |
| Immune mediated hepatitis | 2 (1) | 2 (1) |
| Pancreatitis | 2 (1) | 0 (0) |
| Rash papular | 2 (1) | 0 (0) |
| Arthritis | 1 (0.6) | 1 (0.6) |
| Autoimmune hepatitis | 1 (0.6) | 1 (0.6) |
| Autoimmune pancreatitis | 1 (0.6) | 1 (0.6) |
| Blister | 1 (0.6) | 0 (0) |
| Dermatitis | 1 (0.6) | 0 (0) |
| Diverticulitis | 1 (0.6) | 0 (0) |
| Gastritis | 1 (0.6) | 0 (0) |
| Gastroenteritis | 1 (0.6) | 1 (0.6) |
| Paraparesis | 1 (0.6) | 1 (0.6) |
| Peripheral facial paraparesis | 1 (0.6) | 1 (0.6) |
| Polyarthritis | 1 (0.6) | 1 (0.6) |
| Rash maculo-papular | 1 (0.6) | 1 (0.6) |
| Skin exfoliation | 1 (0.6) | 0 (0) |

**Supplementary Table S-5. Correlation between specific autoantibodies and associated irAEs.**

^a)^ Chi-squared test (Fisher’s exact test for small n, as indicated).

BP, boost phase; IP, induction phase; MT, Maintenance; irAE, immune-related adverse event; irAE(+), patients with at least one irAE; irAE(-), patients without irAE

| **Autoantibody** | **Associated irAE** | **Time point** | **irAE (+)/(-)** | **n_total_** | **Autoantibody-positivity** n (% of n_total_) | **Autoantibody-negativity** n (% of n_total_) | **p value** ^a)^ |
| --- | --- | --- | --- | --- | --- | --- | --- |
| PCA | Gastritis, stomatitis mucosal inflammation, gastroenteritis | IP1 | irAE(+) | 7 | 0 (0) | 7 (100) | 0.516 |
|  |  |  | irAE(-) | 123 | 7 (6) | 116 (94) |  |
|  |  | BP1 | irAE(+) | 5 | 1 (20) | 4 (80) | 0.806 |
|  |  |  | irAE(-) | 82 | 13 (16) | 69 (84) |  |
|  |  | MT | irAE(+) | 2 | 0 (0) | 2 (100.0) | 0.400 |
|  |  |  | irAE(-) | 53 | 14 (26) | 39 (74) |  |
| xANCA | Colitis, diverticulitis, immune-mediated enterocolitis | IP1 | irAE(+) | 16 | 1 (6) | 15 (94) | 0.152 |
|  |  |  | irAE(-) | 154 | 2 (1) | 152 (99) |  |
|  |  | BP1 | irAE(+) | 10 | 0 (0) | 10 (100) | 0.566 |
|  |  |  | irAE(-) | 94 | 3 (3) | 91 (97) |  |
|  |  | MT | irAE(+) | 5 | 1 (20) | 4 (80) | 0.133 |
|  |  |  | irAE(-) | 50 | 2 (4) | 48 (96) |  |
|  | Diarrhea, constipation | IP1 | irAE(+) | 43 | 1 (2) | 42 (98) | 0.747 |
|  |  |  | irAE(-) | 127 | 2 (2) | 125 (98) |  |
|  |  | BP1 | irAE(+) | 29 | 1 (3) | 28 (97) | 0.831 |
|  |  |  | irAE(-) | 75 | 2 (3) | 73 (97) |  |
|  |  | MT | irAE(+) | 16 | 0 (0) | 16 (100) | 0.254 |
|  |  |  | irAE(-) | 39 | 3 (8) | 36 (92) |  |
|  | Pancreatitis and/or autoimmune pancreatitis | IP1 | irAE(+) | 3 | 0 (0) | 3 (100) | 0.815 |
|  |  |  | irAE(-) | 167 | 3 (2) | 164 (98) |  |
|  |  | BP1 | irAE(+) | 1 | 0 (0) | 1 (100) | 0.863 |
|  |  |  | irAE(-) | 103 | 3 (3) | 100 (97) |  |
|  |  | MT | irAE(+) | 0 | 0 (0) | 0 (0) | - |
|  |  |  | irAE(-) | 55 | 3(5) | 52 (95) |  |
| SMA | Hepatotoxicity, immune mediated hepatitis | IP1 | irAE(+) | 4 | 1 (25) | 3 (75) | 0.899 |
|  |  |  | irAE(-) | 130 | 29 (22) | 101 (78) |  |
|  |  | BP1 | irAE(+) | 2 | 0 (0) | 2 (100) | 0.288 |
|  |  |  | irAE(-) | 88 | 32 (36) | 56 (64) |  |
|  |  | MT | irAE(+) | 1 | 1 (100) | 0 (0) | 0.044 (Fisher’s exact 0.200) |
|  |  |  | irAE(-) | 54 | 10 (19) | 44 (81) |  |
|  | Liver value increases (alanine/aspartate aminotransferases) | IP1 | irAE(+) | 16 | 4 (25) | 12 (75) | 0.789 |
|  |  |  | irAE(-) | 118 | 26 (22) | 92 (78) |  |
|  |  | BP1 | irAE(+) | 10 | 1 (10) | 9 (90) | 0.073 |
|  |  |  | irAE(-) | 80 | 31 (39) | 49 (61) |  |
|  |  | MT | irAE(+) | 8 | 2 (25) | 6 (75) | 0.702 |
|  |  |  | irAE(-) | 47 | 9 (19) | 38 (81) |  |
| ANA | Pruritus | IP1 | irAE(+) | 50 | 7 (14) | 43 (86) | 0.451 |
|  |  |  | irAE(-) | 120 | 12 (10) | 108 (90) |  |
|  |  | BP1 | irAE(+) | 33 | 1 (3) | 32 (97) | 0.414 |
|  |  |  | irAE(-) | 71 | 5 (7) | 66 (93) |  |
|  |  | MT | irAE(+) | 23 | 5 (22) | 18 (78) | 0.562 |
|  |  |  | irAE(-) | 32 | 5 (16) | 27 (84) |  |
|  | Rash, Erythema, Skin exfoliation, Blister, Dermatitis | IP1 | irAE(+) | 56 | 6 (11) | 50 (89) | 0.893 |
|  |  |  | irAE(-) | 114 | 13 (11) | 101 (89) |  |
|  |  | BP1 | irAE(+) | 38 | 1 (3) | 37 (97) | 0.298 |
|  |  |  | irAE(-) | 66 | 5 (8) | 61 (92) |  |
|  |  | MT | irAE(+) | 25 | 6 (24) | 19 (76) | 0.307 |
|  |  |  | irAE(-) | 30 | 4 (13) | 26 (87) |  |
|  | Peripheral facial paresis, paraparesis | IP1 | irAE(+) | 3 | 1 (33) | 2 (67) | 0.219 |
|  |  |  | irAE(-) | 167 | 18 (11) | 149 (89) |  |
|  |  | BP1 | irAE(+) | 2 | 0 (0) | 2 (100) | 0.724 |
|  |  |  | irAE(-) | 102 | 6 (6) | 96 (94) |  |
|  |  | MT | irAE(+) | 2 | 0 (0) | 2 (100) | 0.497 |
|  |  |  | irAE(-) | 53 | 10 (19) | 43 (81) |  |
|  | Arthralgia, Myalgia, Musculoskeletal pain | IP1 | irAE(+) | 36 | 4 (11) | 32 (89) | 0.989 |
|  |  |  | irAE(-) | 134 | 15 (11) | 119 (89) |  |
|  |  | BP1 | irAE(+) | 20 | 1 (5) | 19 (95) | 0.870 |
|  |  |  | irAE(-) | 84 | 5 (6) | 79 (94) |  |
|  |  | MT | irAE(+) | 17 | 5 (29) | 12 (71) | 0.149 |
|  |  |  | irAE(-) | 38 | 5 (13) | 33 (87) |  |
|  | Polyarthritis | IP1 | irAE(+) | 4 | 0 (0) | 4 (100) | 0.473 |
|  |  |  | irAE(-) | 166 | 19 (11) | 147 (89) |  |
|  |  | BP1 | irAE(+) | 3 | 0 (0) | 3 (100) | 0.664 |
|  |  |  | irAE(-) | 101 | 6 (6) | 95 (94) |  |
|  |  | MT | irAE(+) | 3 | 0 (0) | 3 (100) | 0.401 |
|  |  |  | irAE(-) | 52 | 10 (19) | 42 (81) |  |
| Anti-TPO/anti-TG | Hypothyreoidism, Hyperthyreoidism, Thyroid-stimulating hormone increase/ decrease | IP1 | irAE(+) | 27 | 1 (4) | 26 (96) | 0.404 |
|  |  |  | irAE(-) | 143 | 2 (1) | 141 (99) |  |
|  |  | BP1 | irAE(+) | 17 | 2 (12) | 15 (88) | 0.063 |
|  |  |  | irAE(-) | 87 | 2 (2) | 85 (98) |  |
|  |  | MT | irAE(+) | 12 | 3 (25) | 9 (75) | 0.007  (Fisher’s exact 0.029) |
|  |  |  | irAE(-) | 43 | 1 (2) | 42 (98) |  |

**Supplementary Table S-6.** **Best overall response (BOR) for nivolumab ± nivolumab+ipilimumab boosts (TITAN-RCC approach).**

| **n (%)** | **All patients** n=170 |
| --- | --- |
| Objective response rate | 59 (35) |
| Complete response | 12 (7) |
| Partial response | 47 (28) |
| Stable disease | 45 (26) |
| Progressive disease | 62 (36) |
| Not evaluable ^b)^ | 4 (2) |

**Supplementary Figure S-1: Study design and time points of sample collection.**

BP1-4, time points of sample collection for assessment of auto antibodies during “boost” phase; CR, complete response; ipi, ipilimumab; IP1-3, time points of sample collection for assessment of auto antibodies during induction phase; MT, time point of sample collection for assessment of auto antibodies during maintenance phase; n, number of serum samples available at the respective time point; nivo, nivolumab; PD, progressive disease; PR, partial response; SD, stable disease.
